# Myelin like electrogenic filamentation and Liquid Microbial Fuel Cells Dataset

**DOI:** 10.1016/j.dib.2022.108447

**Published:** 2022-07-11

**Authors:** Emilio D'Ugo, Lucia Bertuccini, Francesca Spadaro, Roberto Giuseppetti, Francesca Iosi, Fabio Santavenere, Fausto Giuliani, Milena Bruno, Nicola Lovecchio, Silvia Gioacchini, Paola Bucci, Emilia Stellacci, Antonietta Bernardo, Arghya Mukherjee, Fabio Magurano

**Affiliations:** a Department of Infectious Diseases, Istituto Superiore di Sanità, Rome, Italy; b Core Facilities, Istituto Superiore di Sanità, Rome, Italy; c National Center for Innovative Technologies in Public Health, Istituto Superiore di Sanità, Rome, Italy; d Department of Information Engineering, Electronics and Telecommunications, Sapienza, University of Rome, Rome, Italy; e Department of Oncology and Molecular Medicine, Istituto Superiore di Sanità, Rome, Italy; f National Center for Research and Preclinical and Clinical Evaluation of Drugs, Istituto Superiore di Sanità, Rome, Italy; g Department of Food Biosciences, Teagasc, Moorepark, Fermoy, Co. Cork, Ireland

**Keywords:** Electrogenic biofilm, Microbial fuel cells, Filamentation, Myelin-like filaments, Myelin basic protein, Hydrocarbonoclastic biofilm, Microbial evolution, Myelin sheath, **:** L-MFCs, liquid microbial fuel cells, V, voltage, W/m ^2^, power density, A/m^2^, current density, Ω, Ohm, SEM, scanning microscopy, TEM, transmission, CLSM, confocal laser scanning microscopy, MBP, myelin basic protein, O**_4_**, claudin 11, Rp, product resistance, M9, medium, 16S, ribosomal gene, FM 1-43 dye, N-3-triethylammoniumpropyl-4-4-dibutylamino styryl pyridinium dibromide, LB, Luria-Bertani broth, RT, room temperature, OD, optical density, PCR, polymerase chain reaction, rRNA, ribosomal ribonucleic acid, SRA, sequence read archive, SOP, standard operating procedure, PMMA, polymethylmethacrylate, PVC, polyvinylchloride, ABS, acrylonitrile-butadiene-styrene, HMDS, hexamethyldisilazane, DAPI dye, 2-[4-(aminoiminomethyl)phenyl]-1H-indole-6-carboximidamide hydrochloride, W/m2, watts per meter square (power density), SEM, scanning electron microscopy, TEM, transmission electron microscopy

## Abstract

Biofilm at water-oil interface of hypoxic water columns of microcosms, prepared from a lacustrine sample, that used diesel as a carbon source was found to show electrogenic properties. These microcosms named, Liquid Microbial Fuel Cells (L-MFCs) were electrically characterized using a custom electronic analyzer; accurate determination of voltage (V), power density (W/m 2), and current density (A/m2) for both charge and discharge phases was carried out. The instrument made it possible to carry out cell characterizations using resistive loads between 0 Ω (Ohm) and 10 kΩ. During the hypoxic and electrogenic phase, the synthesis of a system of “bacterial piping induction”, produced filaments of hundreds of micrometers in which the microbial cells are hosted. Ultrastructural microscopy collected by scanning (SEM), transmission (TEM), immunofluorescence, Thunder Imager 3D, confocal laser scanning (CLSM) microscopy revealed a “myelin like“ structure during filamentation processes; this “myelin like” structure exhibited cross-reactivity towards different epitopes of the myelin basic protein (MBP) and Claudin 11 (O4) of human oligodendrocytes. The disclosure of these filamentation processes could be helpful to describe further unconventional microbial structures in aquatic ecosystems and of the animal world.

The data that support the findings of this study are openly available in at https://data.mendeley.com/datasets/7d35tj3j96/1.

## Graphical abstract

**Figure:**
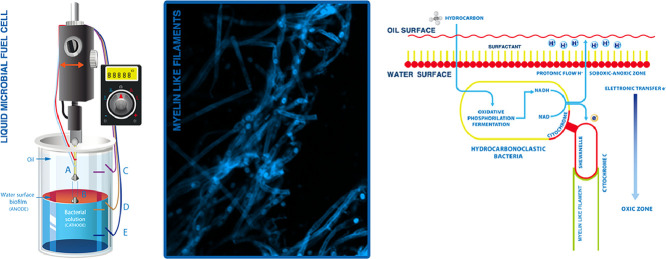

## Specifications Table

**Table:** 

Subject	Environmental Science
Specific subject area	Microbial adaptation to environmental pollution, specifically petroleum hydrocarbon contamination, in freshwater ecosystems. Filamentation process as a response to freshwater hypoxia.
Type of data	Image Graph Figure
How data were acquired	Custom electronic analyzer specific for microbial fuel cells Microscope: SEM, TEM, Thunder Imager 3D, Confocal Laser Scanning Microscopy (CLSM).
Data format	Raw Analyzed Filtered
Parameters for data collection	For L-MFC, RP measurements were performed at a steady state. The average reading of 10 microcosms was considered for data collection. The data collection of electron microscopy, CLSM in triplicate.
Description of data collection	An electronic analyzer specific for microbial fuel cells was used to obtain an electrical characterization of the L-MFCs. For immunofluorescence analysis CLSM signals from different fluorescent probes were acquired in sequential scan settings to avoid aspecific cross-talk. Several filaments were analyzed, and representative results are shown. All the raw data relating to the Microbial Fuel Cells figures, biofilm, metagenomic next generation sequencing data and electrogenic filamentation are reported in the repository created for this article.
Data source location	Istituto Superiore di Sanità Rome Italy Latitude 41.9043 Longitude 12.5181
Data accessibility	Repository name: D'Ugo, Emilio; Mukherjee, Arghya; Spadaro, Francesca; bertuccini, lucia; Iosi, Francesca; Giuseppetti, Roberto; Magurano, Fabio (2022), “Myelin like electrogenic filamentation and Liquid Microbial Fuel Cells Dataset overview.”. https://data.mendeley.com/datasets/7d35tj3j96/1 https://figshare.com/s/72bc73ae14011dc7920d
Related research article	Electrogenic and hydrocarbonoclastic biofilm at the oil-water interface as microbial responses to oil spill 10.1016/j.watres.2021.117092

**Value of the Data**

• Data provides information on new microbial oxidative mechanisms in the water column during oxygen scarcity and in the presence of petroleum hydrocarbons. Petroleum hydrocarbons stimulated the growth of hydrocarbonoclastic floating biofilms capable of creating organic batteries called L-MFC, with the anodic region in the oil-water interface and the cathodic one along the water column.
• Researchers employed in alternative energy and aquatic ecosystems and evolutionary researchers can benefit from this data.
• A new microbial combustion battery (Liquid Microbial Fuel Cell) is described in the dataset; this could represent a starting point for the design of new batteries and new studies on energy flow in aquatic ecosystems. The formation of an electrogenic and hydrocarbon biofilm in the oil-water interface could represent an alternative method for the development of equipment for the removal of oily pollutants from water and in industrial wastewater purification plants. Furthermore, the cross-reactivity of antibodies against vertebrate myelin to myelin-like bacterial structures is shown. This information could be helpful in studying the evolution of myelination.

## 1 Data Description

**Fig. 1:**
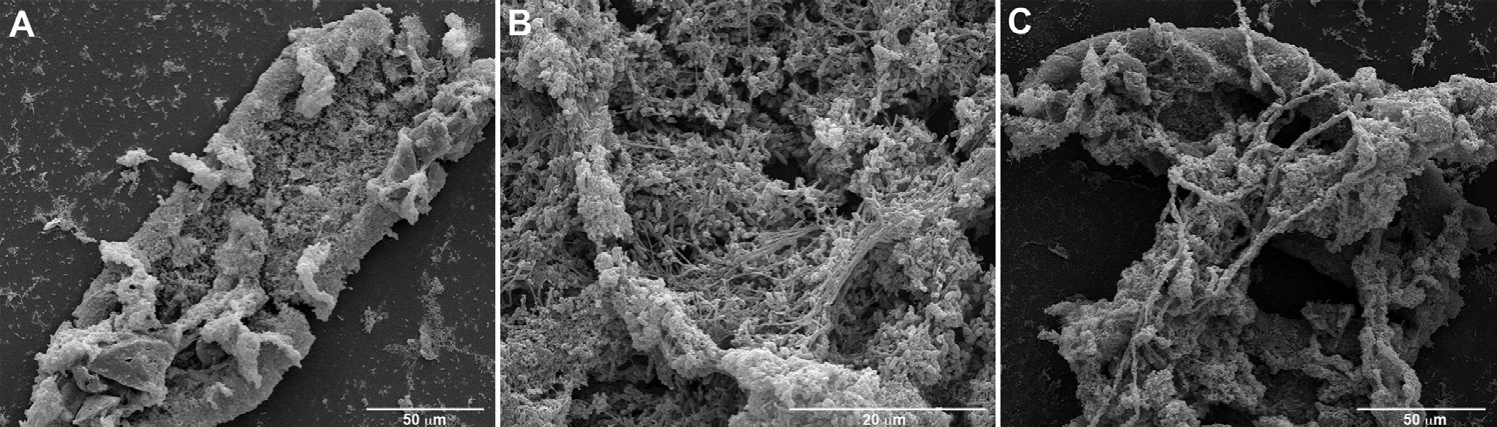
**At the interface of water-diesel microcosms, a planktonic and filamentous biofilm was observed. A**, SEM micrograph of a floating aggregate of bacterial biofilm from the oil-water interface; **B**, the floating biofilm analyzed at high magnification: one type of filamentous bacteria that constitute a sort of irregular biofilm scaffold; **C**, a different kind of filamentous bacteria composed of barrel-shaped cells contained in a large sheath and connecting thick regions of the floating biofilm.

**Fig. 2:**
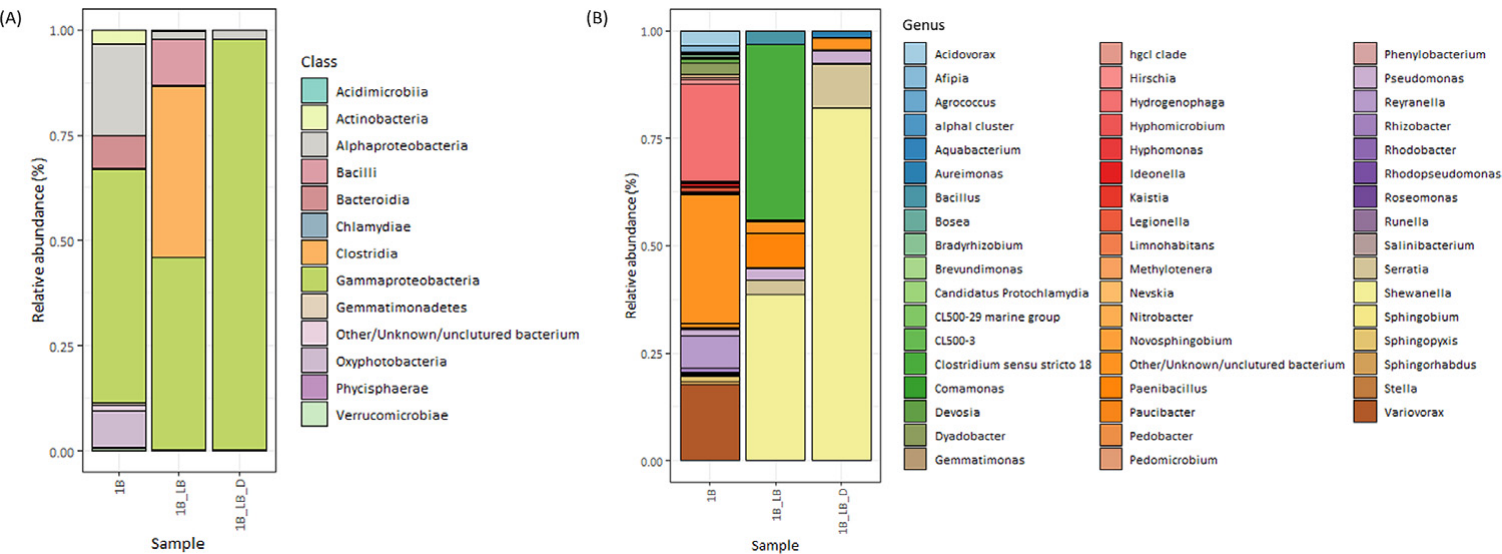
**Microbiome composition of microcosms.** Metagenomic profile of 1B, 1B_LB, and 1B_LB_D samples at the class level. **B**, Taxonomic profile of surface sample obtained from microcosms using diesel as a sole energy source (1B_LB_D) at the genus level as identified at a relative abundance >1%. Ribosomal 16S gene analysis suggests the presence of electrogenic taxa (*Shewanella* sp.).

**Fig. 3:**
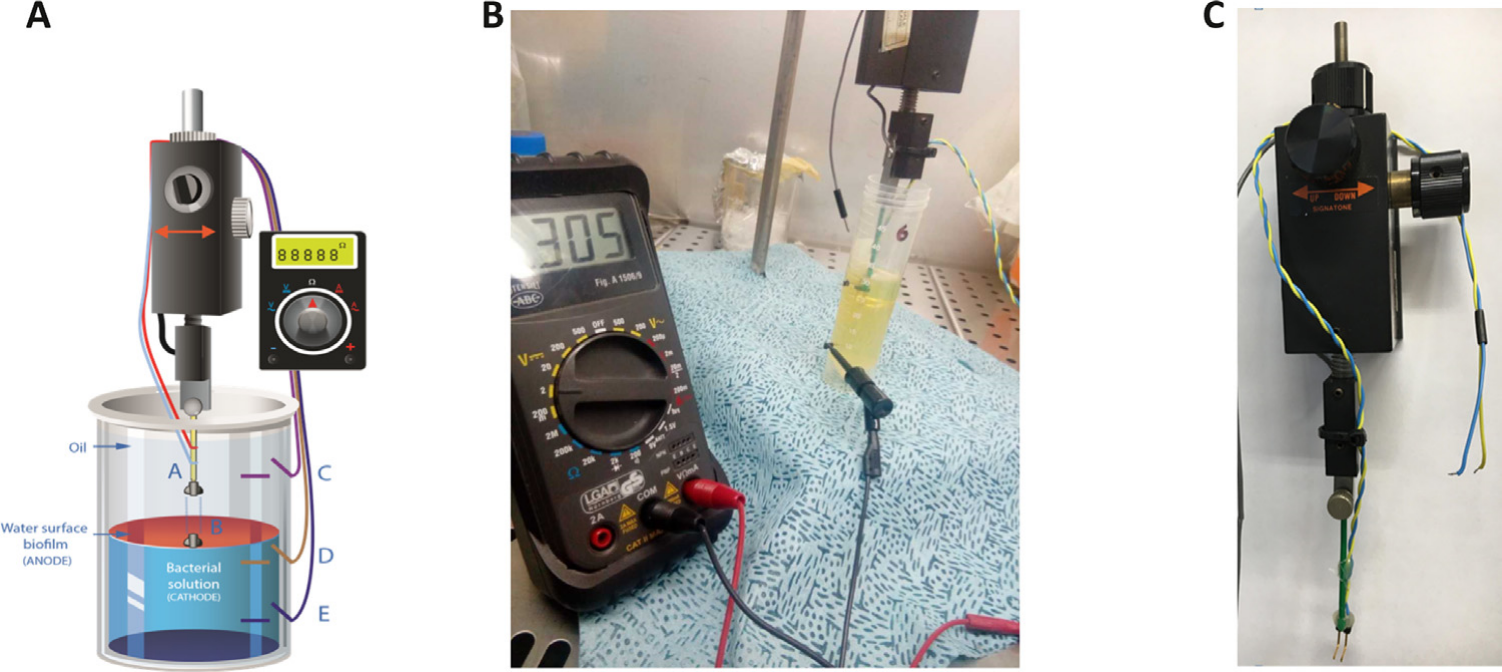
**To verify the electrogenicity of microcosms, Liquid Microbial Fuel Cells (L-MFCs) were developed.** Liquid-Microbial Fuel Cells set-up. Panel **A** shows the L-MFC obtained from falcon tube by introducing gold electrodes on the walls (C, D, E). A micropositioner and a multimeter or other instrument have been used for the detection of redox potential (RP), current (i) and power (W). L-MFC produced current only by placing the electrode of the micropositioner in contact with the membrane (Anode, position B) while positioning it in diesel did not generate electrical signals (position A). The panels **B** and **C** show a multimeter and a Signatone micro-positioner (modified from: doi.org/10.1016/j.watres.2021.117092).

**Fig. 4:**
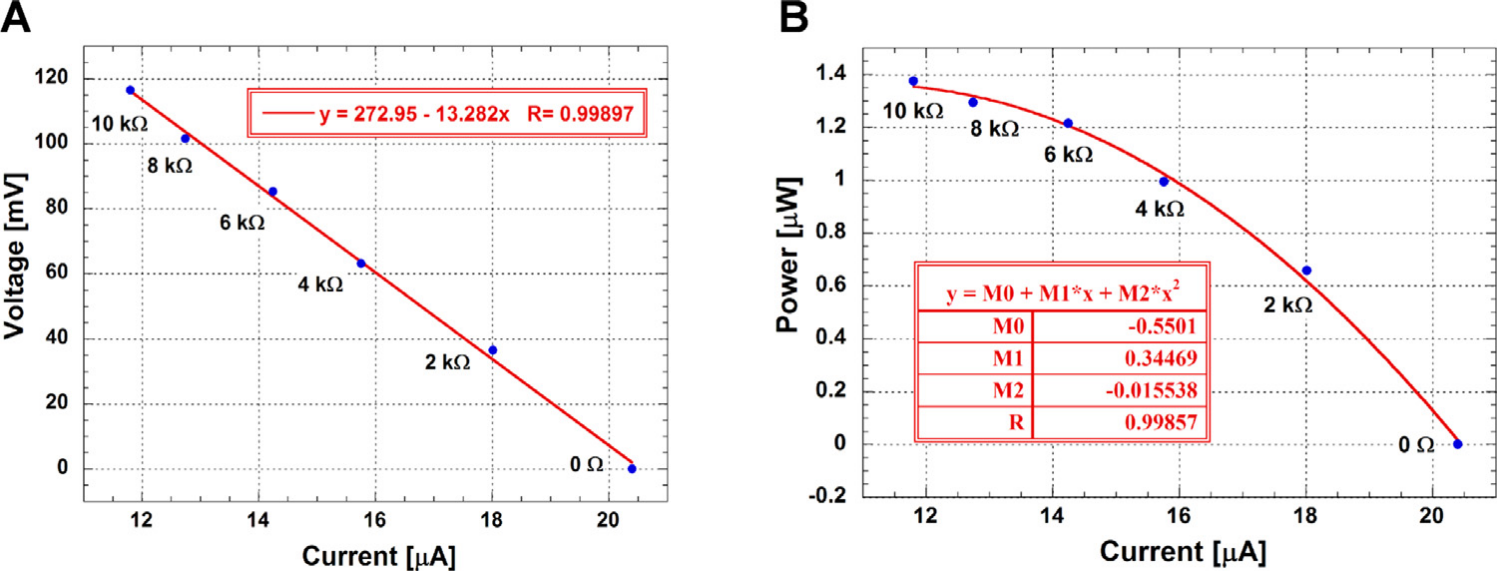
**To characterize L-MFCs, a custom analyzer was used for the polarization curve.** Polarization **(A, modified from D'Ugo** et al. **[])** and power **(B)** curves of the Liquid Microbial Fuel Cell (L-MFC). The blue symbols refer to the experimental data, while the blue equations and lines represent the fitting curves of the results. The area of the anode electrode is 1.1 cm^2^. These graphs are obtained performing a real-time measurement constituted by charge and discharge phases, alternatively. In particular, a set of 6 resistive loads have been used, including 10 kΩ, 8 kΩ, 6 kΩ, 4 kΩ, 2 kΩ and 0 Ω. The duration of each charging phase is 2 min, which is sufficient again to reach the cell's pseudo-steady-state after the discharge phase, while the duration of each discharge is 0.5 s. time[s] 121.07, 242.30, 363.31, 484.42, 605.60, 726.66 voltage[mV] 116.51, 101.53, 85.310, 63.120, 36.489, 0.0000. current[uA] 11.801, 12.743, 14.248, 15.754, 18.013, 20.401 resistance[Ohm] 9872.8, 7968.1, 5987.3, 4006.5, 2025.7, 0.0000 power[uW] 1.3750, 1.2938, 1.2155, 0.99441, 0.65728, 0.0000.

**Fig. 5:**
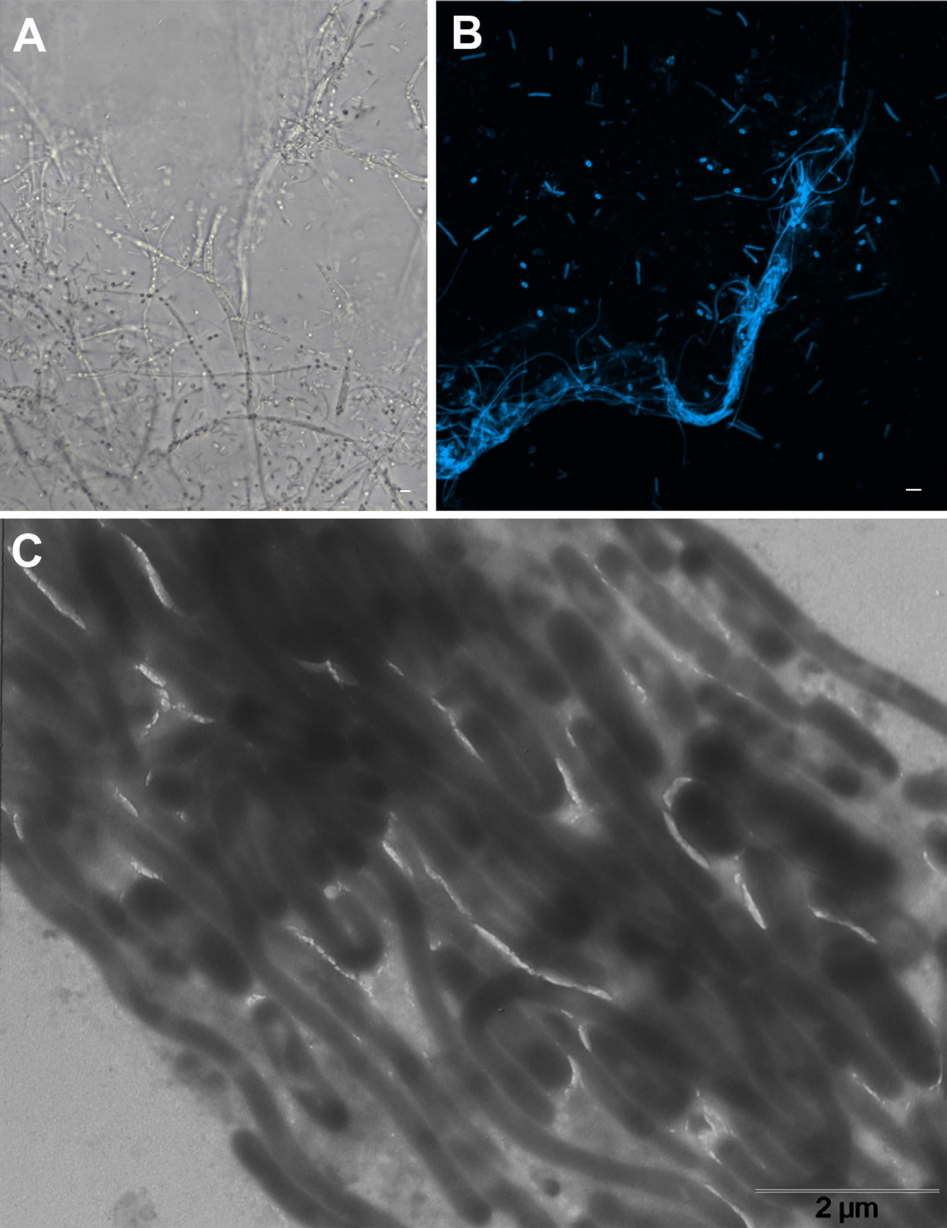
**In the water column of L-MFCs, filamentous structures were observed.** Filamentous structures were observed in the water column of a one-month-old L-MFC. A: Phase contrast image showing abundant filaments with both bacillary and spore-like structures within; B: sampling in the water column of the same culture, which highlights bundle of filaments strictly adherent to each other and free spores. DAPI was used to stain DNA, detected in blue; C: TEM negative staining of a bundle of bacterial filaments. Scale bar 2 µm.

**Fig. 6:**
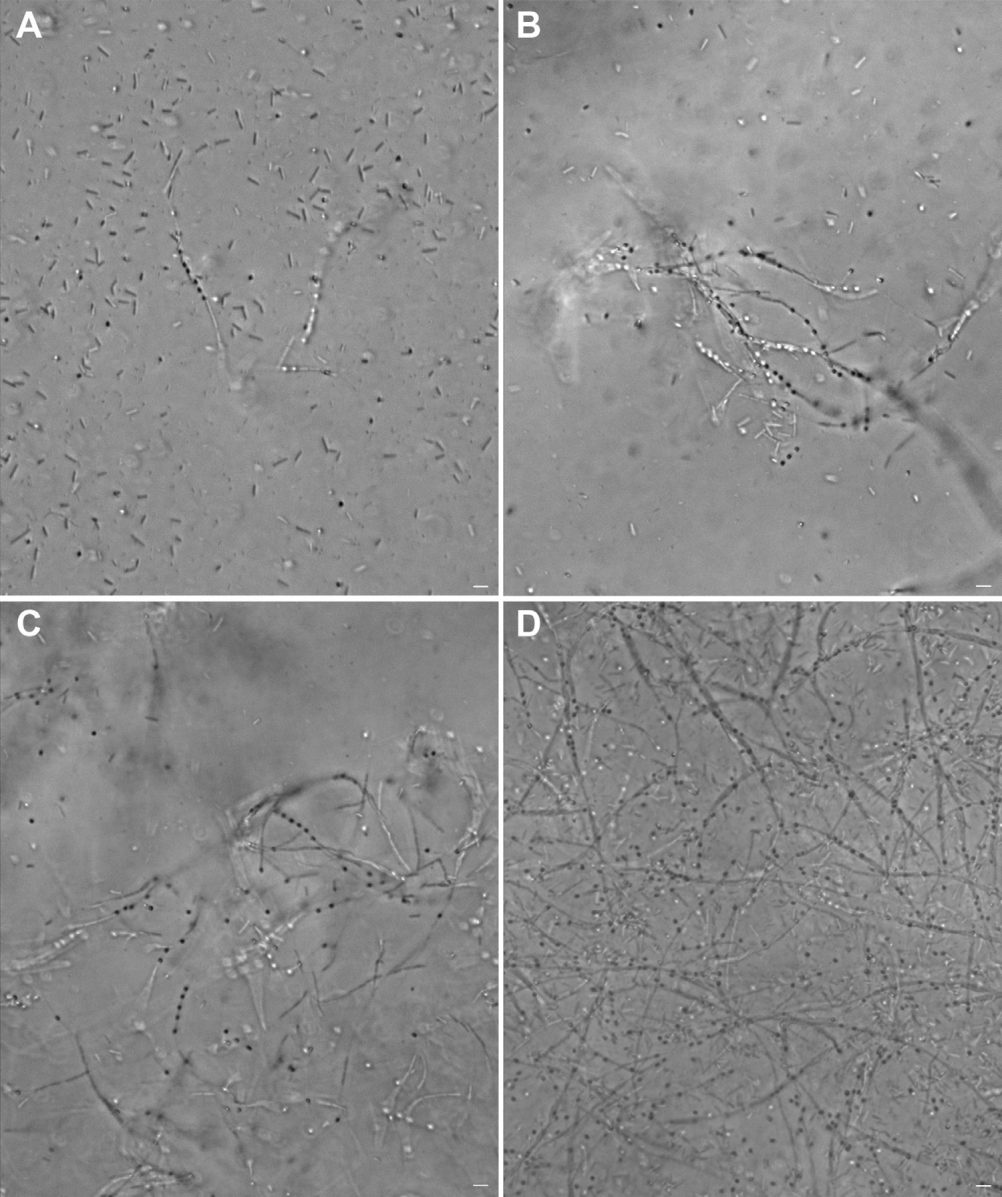
**Anoxic conditions triggered filamentation processes.** Progression of bacterial filaments growth under oxygen-deficient conditions (≤ 3 mg/L). The filaments show an innate fragility of the envelope that leaves spore-like forms in the mean at each sampling (A–C). The enrichment of a filamentous form containing spore-like structures is particularly evident in D (one-month culture). A: 48 h; B: 72 h; C: 7 d. Scale bar 2 µ.

**Fig. 7:**
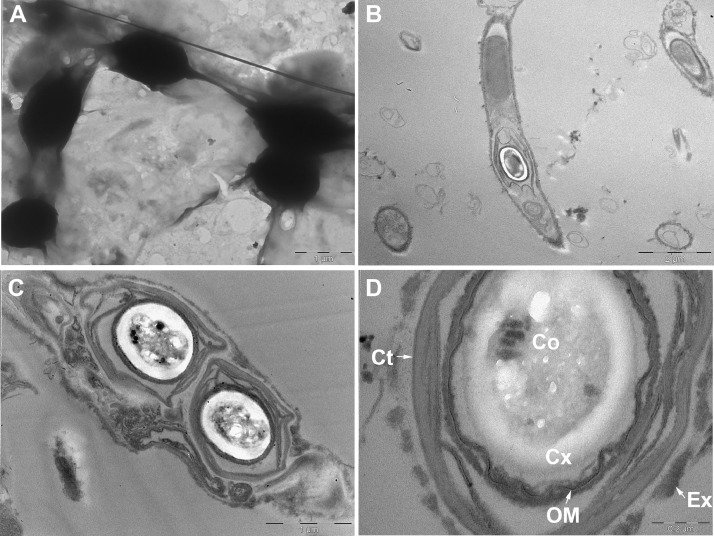
**TEM analysis of bacterial cells of filaments revealed a “myelin like” multi-sheet composition.** A: TEM negative staining of a filament containing spore-like structures in the envelope; B: TEM ultrathin section showing a bacillary and spore-like form in the same envelope; C: TEM micrograph of a train of spores in the same shell; D: TEM high magnification details of a spore-like structure showing a “myelin-like” multi-sheets. Ex: exosporium; Ct: coat; OM: outer membrane; Cx: cortex; Co: core.

**Fig. 8:**
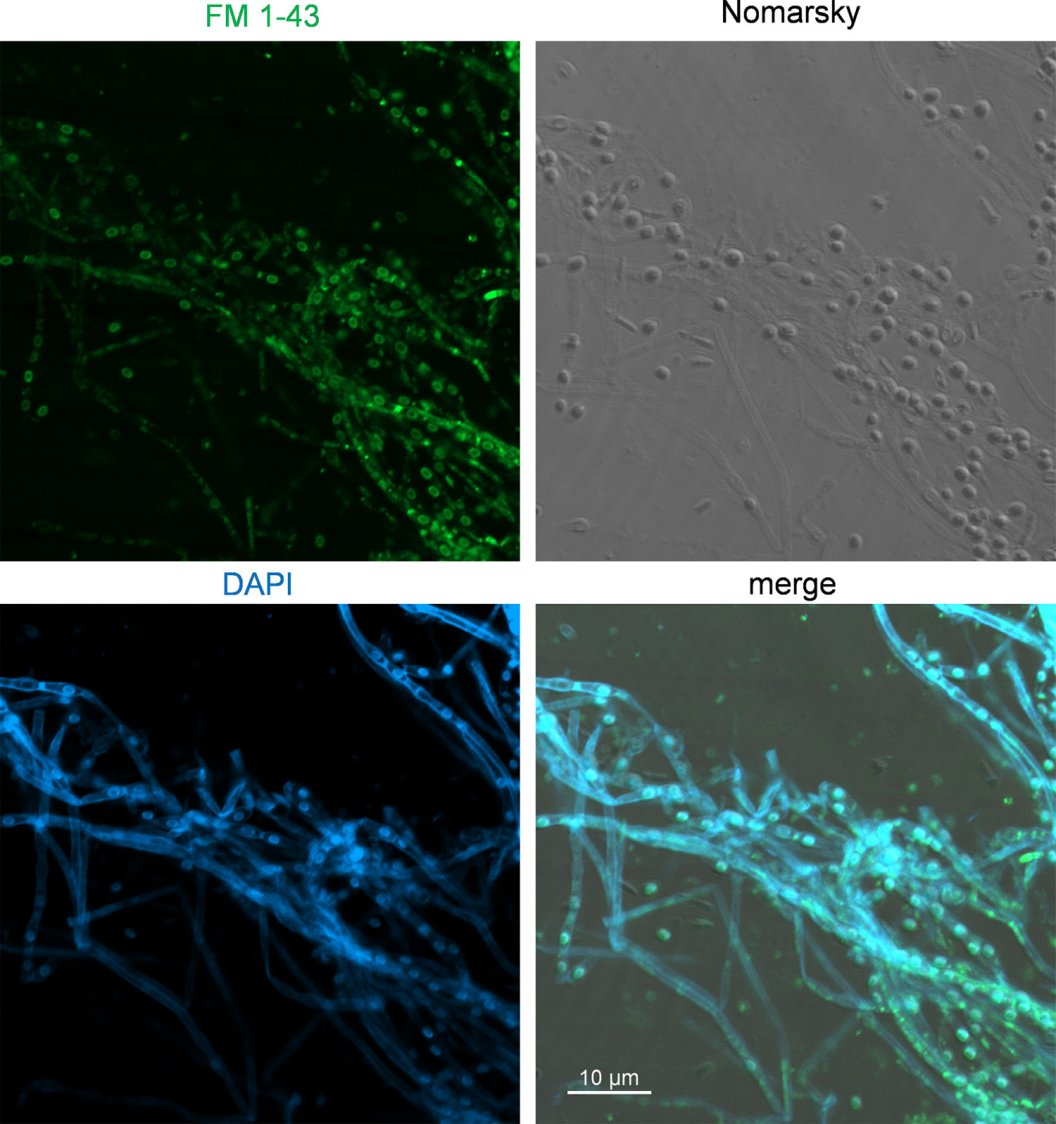
**CLSM analysis revealed lipid contents of filaments (FM 1-43).** Filaments were stained with FM1-43 (green) and DAPI (blue) without fixation and were quickly examined by CLSM. The image shows the bright FM1-43 fluorescence in the spores and the weak signal in the bacilli. Nomarsky image and its overlay with DAPI and FM1-43 are also shown. Panels are representative of 3 independent experiments. Scale bar, 10 µm.

**Fig. 9:**
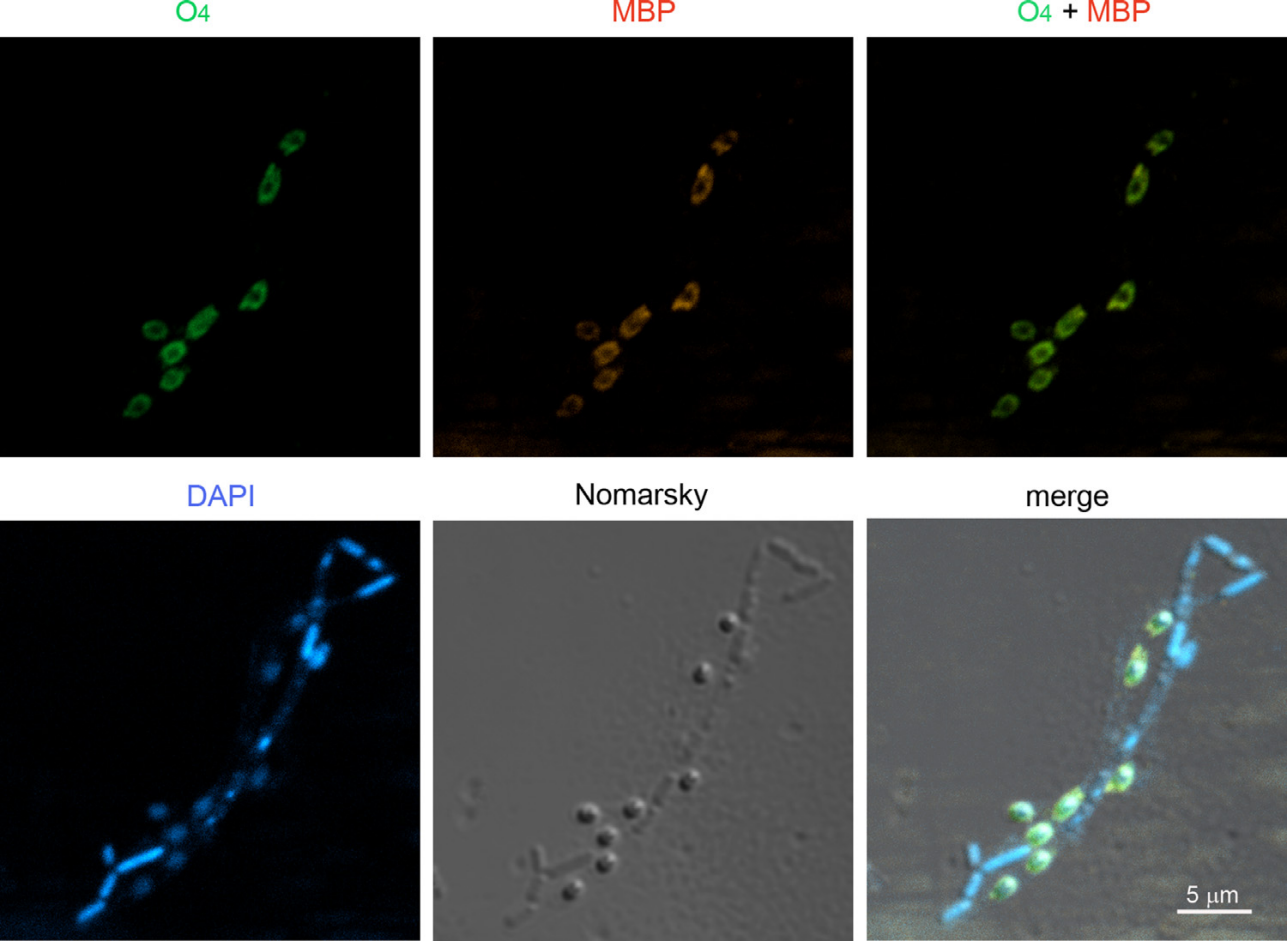
**CLSM revealed cross-reactivity with oligodendrocyte proteins of vertebrates as Myelin Basic Protein (MBP) and O_4_.** MBP and O**_4_** (Claudin 11) detection in myelin-like filaments of the enriched sample. Filaments from the L-MFC water column were fixed and then stained with anti-MBP (orange) and O4 (green) antibodies. DAPI was used for DNA staining (blue). Co-localization of MBP and O4 proteins on the membrane of the spores and the absence of signal on the bacilli are shown in merged images. Scale bar, 5 µm.

Ultrafiltered samples collected from Lake Pertusillo were resuspended in M9 medium and incubated at 30 °C, in microcosms with diesel oil as the sole carbon source. The tubes of the microcosms were hermetically sealed in such a way as to generate conditions of microaerophilia. The samples were kept without shaking in order not to disturb the formation of biofilms. At the water-diesel interface in microcosms, a planktonic and filamentous biofilm was observed (Fig. 1). The sequences of ribosomal 16S gene of the microbial community capable of growing under these conditions belong mainly to the class of Gammaproteobacteria. In the sample grown with diesel as the only source of carbon, more than 90% of the bacteria are sequestered in this class of bacteria with versatile metabolic potential (Fig. 2, Supplementary data, Table 1 class level). Microbiome composition of biofilm microcosms at genus level suggested the presence of electrogenic taxa like *Shewanella sp* (Fig. 2, Supplementary data: Table 1 genus level). The evidence of *Shewanella sp* and filamentous structure in the planktonic biofilm suggest to verify the electrogenicity of microcosms. In particular, Liquid Microbial Fuel Cells (L-MFC) have been developed by modifying the tubes (Falcon, 50 ml tubes) from which the biofilm originates (Fig. 3A, B, Supplementary Fig. 1). The use of a micropositioner (Fig. 3C) with an electrode that could be positioned on the growing biofilm, made possible micromovements of the electrode, to test the electrogenicity of the biofilm. By placing the electrode of the micropositioner in parts other than the biofilm, it did not produce electrogenicity. In order to obtain a complete electrical characterization of the L-MFCs, a custom electronic analyzer dedicated to microbial fuel cells was used: polarization and power curves were determined (Fig. 4 A, B). A prototype L-MFC was analysed to explore the presence, along with water column, of microbial filaments: filamentous structures were observed up to distances of 6-7 cm from the biofilm (Fig. 5). In order to study the filamentation processes, different oxygen concentrations and their correlations with filaments formation were analysed during bacterial growth. The filaments were increasingly evident below 3.5 mg /L of oxygen. Below these oxygen values, filaments with rounded bacterial spore-like cells appeared in the samples. (Fig. 6 A–D). TEM analysis of these spore like cells revealed a “myelin like” multisheath composition (Fig. 7). CLSM analysis revealed lipid contents of these multisheath structures (Fig. 8, FM 1-43 staining) when stained with a specific dye for biological membranes also neuronal such as FM 1-43. At CLSM and western blot (data not shown) these bacterial spheroidal cells, revealed also cross-reactivity in comparison to internal control (bacillary forms), with oligodendrocyte proteins of vertebrates (Fig. 9) such as myelin basic protein (MBP) and Claudin 11 (O**_4_**).

## 2 Experimental Design

The formation of biofilms on surfaces other than solid ones represents an emerging topic, especially for biofilms that grow between liquids of different densities [1]. The presence of oil layers on the surface of environmental waters poses the problem of how aquatic communities colonize the interface and how they can cope with the anoxic gradients that are produced during the degradation of the oily layer. It is unknown how hydrocarbonoclastic biofilms can ensure a continuous energy flow from the oil-water interface to the water column. This study aimed to investigate lake microbial community adaptations under oily slick simulation (see Graphical Abstract).

## 3 Materials and Methods

### 3.1 Sampling Site and Ultrafiltration

Samples were collected from Madonna Grumentina (N 40.29172, E 15.92957), Lake Pertusillo, situated in the Basilicata region (Italy), on May 22, 2017. Samples were collected three months after algal bloom and oil spill from the nearby oil extraction plant in February 2017 [2,3]. Five liters of water were taken just below the surface and transported to the laboratory, and stored at + 4 °C until the time of analysis, which was carried out within 24 h. The sample was concentrated using a polysulfone hollow fibre with a filtration surface of 1. 8 m2 and a cut-off size of 20 kD module (HF-80S, Fresenius Medical Care, Bad Homburg, Germany). The cartridge has a filter that excludes particles with a diameter of less than 25–30 nm and the collection of those with a larger diameter. The filtration system consists of a filter cartridge with a valve system connected to a peristaltic pump. The concentration consists of a filtration phase in which the microorganisms adhere to the filter and an elution phase in which the microorganisms are resuspended through a reverse elution process. Through this system, 5 L of water sample were eluted in 500 mL of elution solution (Backflush solution). Before starting the filtration operation, the elution solution was prepared by adding phosphate buffer (PBS, Sigma) to 1 L of deionized or ultrapure water, bringing the solution to a final concentration of 0.01M. The solution was sterilized at 121 °C for 20 min and allowed to cool to room temperature. Add 5 ml of Tween 80 (Sigma) for a final concentration of 0.5% and 100 mg of sodium hexaphosphate (Napp, Sigma) and 0.01 ml of antifoam B emulsion (Sigma). For this study, we selected sample 1B (Total Hydrocarbon Content of 900 µg/L and algal bloom of Peridinium sp. 10^6^ cells/ mL; [3,4]. The elutriate was obtained by “back flushing” the filter in reverse so that all captured particles, including microorganisms, were released. The elutriate was aliquoted and used for laboratory investigations.

### 3.2 Culture Media and Fuels

Ultrafiltered samples were resuspended in Aretina Acque Minerali (S.N.C., Florence, Italy), filtered with a 0.1 µm filter (Sartorius, Italy) and autoclaved. Alternatively, samples were resuspended freshwater salts solution (M9) prepared as previously described without adding sea salts. After autoclaving, 5 ml of sterile trace element solution were added to each litre of cooling solutions. The trace element solution contained: 30.1 g/L MgSO_4_, 27.3 g/L FeSO_4_ x 7H_2_O, 5.4 g/L MgO, 1.0 g/L CaCO_3_, 0.72 g/L ZnSO_4_ × 7 H_2_O, 0.56 g/L MnSO_4_ × H_2_O, 0.125 g/L CuSO_4_ × 5 H_2_O, 0.14 g/L CoSO_4_ × 7 H_2_O, 0.03 g/L H_3_BO_3_, 0.004 g/L NiCl_2_ × 6 H_2_O, 0.006 g/L Na_2_MoO_2_ × 2 H_2_O and 25.6 mL/L HCl (36%). Diesel was purchased from Italiana Petroli (GO – Diesel Plus), Kuwait Petroleum Italia (Q8), and filtered with a 0.2 µm filter. To assess sterile conditions, tests were performed as suggested by the European Pharmacopoeia and reported elsewhere [4], [5], [6].

### 3.3 Microcosms and Biofilms

An aliquot (5 ml) of filtered environmental sample 1B was enriched in 50 ml of Luria-Bertani (LB) broth for a week at 30 °C (1B_LB). To remove all traces of residual carbon source, the enriched sample was centrifuged at 6000 rcf for 10 min at room temperature (RT) and washed twice with M9. The pellets were resuspended in M9 and estimated by spectrophotometer at 600 nm (SPECTROSTAR nano, BMG LABTECH, Germany). At 50 mL tubes (Falcon) containing 30–40 mL of M9 or Aretina water were added trace elements (5 ml/L) and 10% of diesel as a sole energy source; these were then incubated for six months at 30 °C (sample 1B_LB_D). These microcosms derived from sample 1B_LB were supplemented with LB and 10% diesel oil and incubated at 30 °C to study growth under different nutritional conditions. To evaluate growth, aliquots (10 ml) of samples were pelleted and resuspended in 1 mL M9; optical density (OD) was measured at 600 nm using a spectrophotometer.

### 3.4 Isolation of Total Microbiome DNA and High Throughput 16S rRNA gene Sequencing

Total environmental DNA was extracted with NucleoSpin Tissue Kit (Macherey-Nagel, Germany) following the manufacturer's instructions and quantified with a spectrophotometer (NanoDrop, Thermo Fisher). PCR was performed in a Thermocycler (Perkin Elmer) using the following conditions: 95 °C for 3 min, {95 °C for 30 s, 55 °C for 30 s, and 72 °C for 30 s} for 25 cycles, and a final extension step of 72 °C for 5 min. Primers used were FWD: CCTACGGGNGGCWGCAG and REV: GGGTTGCGCTCGTTGCGGG. High throughput sequencing of the V3-V5 region of the 16S rRNA gene for microbiomes was performed by Eurofins MWG Operon (Ebersberg, Germany). Sequencing was performed using Illumina MiSeq sequencing system using the 2 × 300 bp read modus (v3 chemistry from Illumina). The Sequence data were submitted to the NCBI Sequence Read Archive (SRA) under BioProject number PRJNA412797 (1B: SRX3362329. 1B_LB: SRX3362328. 1B_LB_D: SRX3362331).

### 3.5 Analysis of Microbial Community Structure

All sequences generated in the present study were evaluated for quality using FastQC [7] and processed in mothur [8] with trimming of adapters, primers and barcodes. To do this, the MiSeq SOP for mothur was followed; the SOP can be found here: https://mothur.org/wiki/miseq_sop/. Additional quality control was carried out using the following criteria: maxhomop = 6, maxambig = 0, minlength = 200, qwindowaverage = 30, bdiffs = 1, pdiffs = 2, and tdiffs = 2. Mothur was used to align quality-filtered sequences to the SILVA [9] database. Chimeric sequences were removed using the mothur implementation of vsearch [10], where the sequences were searched against themselves, as described in the SOP. QIIME version 1.9.1 (downloadable here: http://qiime.org/home_static/dataFiles.html) was used for open reference OTU calling and taxonomic assignment of the high-quality 16S rRNA sequences against the SILVA nr v132 release. Taxonomic classification was summarized in QIIME using the *summarize_taxa.py* script and subsequently visualized in R with ggplot2.

### 3.6 Liquid Microbial Fuel Cells (L-MFCs) and Redox Potential, O2 and pH Measurements

To obtain a Liquid Microbial Fuel Cell (L-MFCs), [11], gold electrodes were fixed on the wall of the tubes containing the environmental bacterial solution (cathode) and a pair of bare gold electrodes or gold needle electrodes were immersed and placed on the microbial biofilm that developed in diesel-water interface (anode) . The immersion electrodes were mounted on a Signatone S-725, (Lucas Signatone Corporation, USA) a micro-positioner equipped with micro-adjustment rings and connected to a multimeter. The surface areas of immersive gold electrodes measured 0.55 cm² each. L-MFC set up for the 50 mL tube is shown in Fig. 3 and Supplementary Fig. 1.

To have easy access to the water column of the microcosm during RP, O_2_ and pH measurements, an L-MFC prototype with a radius of 12 cm and a height of 54 was built for this study (Supplementary Fig. 1). The PMMA (polymethylmethacrylate) tube was equipped with two caps of PVC (Polyvinylchloride). The top cap was equipped with six brass nozzles to allow the entry of liquids and the insertion of 0.5 cm diameter pipes to regulate the gas exchanges in the tank. Two holes with adjustable ring nut allowed the sliding and fixing of two hollow steel rods. The bottom cap hole was connected to a brass nozzle for draining the tank. The hollow steel rods at one end were connected to two annular supports in ABS (acrylonitrile-butadiene-styrene) where the electrodes were housed. Each electrode was soldered to the end of a copper wire which was closed and insulated with plastic, inside the hollow steel rods. The copper wires in the upper end were connected to the equipment used for RP measurements. The vertical movement of the hollow steel rods made it possible to move the electrodes in the L-MFC water column to the height desired by the operator. RP readings were obtained by placing one electrode on the biofilm (anode) and the other in the solution containing the bacteria (cathode). For this study two carbon plates of about 4, 6 cm^2^ were used as electrodes for redox measurement. RP measurements were performed at a steady state. A custom electronic analyzer dedicated to microbial fuel cells was also used [12] to obtain a complete electrical characterization of the L-MFCs. This measurement tool allowed complete electrical characterization of the L-MFCs through analysis of the charge and discharge phases of MFCs, with accurate determination of the values of voltage (V), power density (W/m ^2^), and current density (A/m ^2^). The instrument made it possible to carry out cell characterizations using resistive loads between 0 Ω (short circuit measurement) and 10 kΩ (Fig. 4). The dissolved oxygen and pH measurements of the L-MFC prototype were performed by microprocessor HI 9143 (Hanna Instruments, Italy) and HD8602 (Delta OHM, Italy).

### 3.7 Enrichment of Filamentous Bacteria from the Water Column of L-MFC

Bacterial filaments were collected from the water column of the L-MFC prototype (Supplementary Fig. 1A). The filaments were pelleted at 300 rcf and gently resuspended in M9. Filaments were smeared on plates with LB agar and incubated at 30 °C for 24 h in aerophilic conditions. To study the bacterial filamentation, single colonies from plates were taken and grown in LB with 15ml tubes (Falcon) incubating them in micro ventilation conditions at 30 °C for 7–10 d at 0 rpm. The preparations were constantly observed with a ProgResC10 optical microscope (Nikon). Given the difficulty of extracting DNA from the filaments, the individual colonies, capable of producing filaments and redox potentials, were grown under aerobic conditions at 28–30 °C in LB broth for 48–72 h. This prevented the formation of filaments (filament) and allowed to extract the DNA directly from the single cells form. The genome was extracted with the DNA tissue kit (Machery-Nagel) as described above.

### 3.8 Scanning (SEM) and Transmission (TEM) Electron Microscopy

Floating biofilms (1B_LB_D) were collected from the diesel-water interface and left to adhere on poly-lysine treated glass coverslips for 4 h at RT. Samples were processed as previously described with some modifications [13]. Samples were post-fixed by osmium tetroxide for 1h at RT, and dehydrated through a graded series of ethanol solutions until the complete ethanol substitution by hexamethyldisilazane (HMDS)(30 min 1:1 ethanol: HMDS; 1h HMDS). Samples were then air dried under the chemical hood for 2 . Furthermore, a sample enriched in filaments were delicately taken from the water column of the culture tube, left to adhere to poly-lysine treated glass coverslips for 2 h and directly air-dried.

All dried coverslips were fixed on stubs, gold-sputtered (10 nm) and analyzed by FE-SEM Quanta Inspect F (FEI - Thermo Fisher Scientific; Eindhoven - The Netherlands).

The same kind of samples described above for SEM analysis were also processed for TEM as previously described by Perry and Gilbert with slight modifications [14]. Samples were fixed in 2,5% glutaraldehyde, 2% paraformaldehyde, 2 mM CaCl_2_ in 0.1 M sodium cacodylate buffer overnight at 4 °C, post fixed in 1% osmium tetroxide for 1 h, dehydrated by a graded series of ethanol solutions and then embedded in Agar 100 resin (Agar Scientific Ltd, Stansted Mountfitchet - England). Ultrathin sections, obtained by a UC6 ultramicrotome (Leica Microsystem, Wetzlar - Germania), were stained with uranyl acetate and Reynolds’ lead citrate.

Bacterial filaments sampled in the central part of the broth-oil tube culture were also observed by negative staining method by ammonium molybdate 2% staining solution.

All TEM samples were examined by EM 208S TEM (FEI - Thermo Fisher Scientific; Eindhoven - The Netherlands) at 100 kV, equipped with the Megaview SIS camera (Olympus).

### 3.9 Immunofluorescence, Thunder Imager 3D Cell Culture and Confocal Laser Scanning Microscopy (CLSM)

Thunder Imager 3D cell culture microscope (Leica Microsystem, Wetzlar – Germany) was used to analyze the large filamentous structures observed in cell aggregates 1B_LB_D. Cells were first stained in 96-well microplates with 10 μg/mL 4’,6-diamidino-2-phenylindole (DAPI, Thermo Fisher Scientific, Invitrogen) for 10 min at room temperature (RT) and immediately analyzed under the microscope without fixation. Images were acquired by using a 40x/0.60 NA objective and a Stage Navigator, that allowed to acquire a large area by combining multiple fields of view (Image Stiching).

For bacterial cell membrane detection the filamentous structures were also examined at high resolution by CLSM. In detail, filaments from the enriched sample were first stained in the culture plate with DAPI for 10 min at RT, followed by FM1-43 (5 μg/mL; Thermo Fisher Scientific, Invitrogen) for 5 min at RT. After staining, bacterial cells were seeded on a microscope slide with a broken tip to avoid the breakage of filaments, covered with a cover glass and quickly examined by CLSM.

In order to reveal the expression of Myelin Basic Protein (MBP) and Claudin 11 (O_4_) proteins on the filaments with both bacillary and spore-like structures, bacterial cells were collected by centrifugation at 6000 rcf for 15 s. Bacteria were seeded on a glass slide and fixed with 4% paraformaldehyde for 30 min at RT. Cells were then incubated for 2 h with the oligodendroglia marker O_4_ (hybridoma supernatants IgM, custom made, also called Claudin 11) or with different types of anti-MBP antibodies: (1) a mouse monoclonal anti-MBP (1:100, #MAB382, from Chemicon International, USA), (2) a.a. 129-138 (GTLSKIFKL), an anti-Myelin Basic Protein SMI 94R, (3) a.a. 70-89 (PVVHFFKNIVTPRTPPPSQ) from Covance, MA, and (4) a rabbit polyclonal a.a. 102–116 (GGRASDYKSAHKGF) from Sigma-Aldrich, Italy [15]. As secondary antibodies, fluorescein-conjugated goat anti-mouse IgM or Cy^TM3^-conjugated goat anti-mouse or goat anti-rabbit IgG were used for anti-O4 or anti-MBP primary antibodies, respectively (1:200, Jackson ImmunoResearch Laboratories, Inc., West Grove, PA). In some experiments, filaments with both bacillary and spore-like structures were double stained for O4 and MBP detection with the appropriate combination of antibodies, by using the mouse monoclonal anti-O4 and the rabbit polyclonal anti-MBP as primary antibodies. Coverslips were finally mounted with Vectashield Mounting Medium-plus DAPI (Vector Laboratories, Burlingame, CA).

For CLSM analysis, images were acquired by an LSM980 Zeiss microscope equipped with Airyscan 2, using a 63x/1.40 NA oil objective and excitation spectral laser wavelengths of 405, 488 and 543 nm. The Zeiss Confocal Software Zen 3.0 (Blue edition) was used for image acquisition and processing. Signals from different fluorescent probes were taken in sequential scan settings to avoid the aspecific cross-talk in the emission spectra. Several filament aggregates were analysed in each experimental condition, and representative results are shown.

## Ethics Statement

Not applicable.

## CRediT authorship contribution statement

**Emilio D'Ugo:** Conceptualization, Methodology, Writing – original draft, Visualization, Validation, Writing – review & editing. **Lucia Bertuccini:** Writing – review & editing, Visualization, Validation, Data curation, Investigation. **Francesca Spadaro:** Validation, Investigation, Methodology. **Roberto Giuseppetti:** Validation, Investigation. **Francesca Iosi:** Validation, Investigation. **Fabio Santavenere:** Validation, Investigation. **Fausto Giuliani:** Validation, Investigation. **Milena Bruno:** Validation, Investigation. **Nicola Lovecchio:** Validation, Investigation, Software. **Silvia Gioacchini:** Validation, Investigation. **Paola Bucci:** Validation, Investigation. **Emilia Stellacci:** Validation, Investigation, Conceptualization. **Antonietta Bernardo:** Validation, Investigation, Visualization, Conceptualization. **Arghya Mukherjee:** Writing – review & editing, Validation, Software. **Fabio Magurano:** Supervision, Funding acquisition.

## Declaration of Competing Interest

The authors declare that they have no competing financial interests or personal relationships which have or could be perceived to have influenced the work reported in this article.

## Data Availability

Myelin like electrogenic filamentation and Liquid Microbial Fuel Cells Dataset overview (Original Data) (Mendeley Data).

## Appendix Supplementary materials

Image, application 1
