# Supplementary material for: Myelin like electrogenic filamentation and Liquid Microbial Fuel Cells Dataset
Source: Data Brief. 2022 Jul 11;43:108447. doi: 10.1016/j.dib.2022.108447 (PMC9294656; doi:10.1016/j.dib.2022.108447)
Supplement: Supplementary file 1 [file mmc1.docx]

**Supplementary information**

**Liquid Microbial Fuel Cells (L-MFC) and “Myelin like” filaments Dataset.**

Emilio D’Ugo, Lucia Bertuccini, Francesca Spadaro, Roberto Giuseppetti, Francesca Iosi, Fabio Santavenere, Fausto Giuliani, Milena Bruno, Nicola Lovecchio, Silvia Gioacchini, Paola Bucci, Emilia Stellacci, Antonietta Bernardo, Arghya Mukherjee, and Fabio Magurano

**Table 1 Supplementary information.** Class and Genus taxonomic composition of sample 1B, 1B_LB and 1B_LB_D (Ribosomal 16S RNA gene sequences: Row data).

| make class and genus level figure | Colonna1 | Colonna2 | Colonna3 |
| --- | --- | --- | --- |
|  | **Sample** |  |  |
| #Class level | **1B** | **1B_LB** | **1B_LB_D** |
| Acidimicrobiia | 0,17284024 | 0 | 0 |
| Actinobacteria | 3,34952468 | 0,35903840 | 0,0864927 |
| Alphaproteobacteria | 21,4768900 | 1,82251014 | 2,11907164 |
| Bacilli | 0 | 11,0872619 | 0,10090817 |
| Bacteroidia | 7,777810889 | 0,09366219 | 0 |
| Chlamydiae | 0,24138033 | 0 | 0 |
| Clostridia | 0,07450010 | 40,5713393 | 0 |
| Gammaproteobacteria | 55,4072175 | 45,6954417 | 97,448464 |
| Gemmatimonadetes | 0,75692106 | 0 | 0 |
| Oxyphotobacteria | 8,65989212 | 0,12098033 | 0,2018163 |
| Phycisphaerae | 0,20562028 | 0,00780518 | 0 |
| Verrucomicrobiae | 0,61984086 | 0,10927255 | 0 |
| Other/Unknown/unclutured bacterium | 1,25756176 | 0,13268810 | 0,0432463 |
|  | **Sample** |  |  |
| #Genus level | **1B** | **1B_LB** | **1B_LB_D** |
| Acidibacter | 0,00018 | 0,00000 | 0,00000 |
| Acidiphilium | 0,000060 | 0,000000 | 0,000000 |
| Acidovorax | 0,03516 | 0,00047 | 0,00000 |
| Afipia | 0,014632 | 0,000156 | 0,001009 |
| Allorhizobium-Neorhizobium-Pararhizobium-Rhizobium | 0,000834 | 0,000000 | 0,000000 |
| Amaricoccus | 0,000268 | 0,000000 | 0,000000 |
| Aminobacter | 0,000209 | 0,000000 | 0,000000 |
| Aquabacterium | 0,00161 | 0,00008 | 0,00000 |
| Arenimonas | 0,00054 | 0,00000 | 0,00000 |
| Aureimonas | 0,000417 | 0,000000 | 0,015136 |
| Bauldia | 0,000954 | 0,000000 | 0,000000 |
| Bdellovibrio | 0,00021 | 0,00000 | 0,00000 |
| Bosea | 0,001788 | 0,000000 | 0,000000 |
| Bradyrhizobium | 0,003218 | 0,000000 | 0,000000 |
| Brevundimonas | 0,001639 | 0,000117 | 0,000000 |
| Caenimonas | 0,00051 | 0,00000 | 0,00000 |
| Candidatus Alysiosphaera | 0,000238 | 0,000000 | 0,000000 |
| Candidatus Bealeia | 0,000149 | 0,000000 | 0,000000 |
| Candidatus Berkiella | 0,00057 | 0,00000 | 0,00000 |
| Candidatus Finniella | 0,000060 | 0,000000 | 0,000000 |
| Candidatus Methylopumilus | 0,00027 | 0,00000 | 0,00000 |
| Candidatus Paracaedibacter | 0,000089 | 0,000078 | 0,000000 |
| Candidatus Symbiobacter | 0,00012 | 0,00012 | 0,00000 |
| Caulobacter | 0,000268 | 0,000000 | 0,000000 |
| Cellvibrio | 0,00077 | 0,00000 | 0,00000 |
| Cereibacter | 0,000089 | 0,000000 | 0,000000 |
| Chelatococcus | 0,000060 | 0,000000 | 0,000000 |
| Comamonas | 0,00012 | 0,00137 | 0,00000 |
| Coxiella | 0,00009 | 0,00000 | 0,00000 |
| Defluviimonas | 0,000060 | 0,000000 | 0,000000 |
| Devosia | 0,008106 | 0,000000 | 0,000000 |
| Dokdonella | 0,00012 | 0,00000 | 0,00000 |
| Duganella | 0,00006 | 0,00000 | 0,00000 |
| Ellin6067 | 0,00012 | 0,00008 | 0,00000 |
| Escherichia-Shigella | 0,00018 | 0,00000 | 0,00000 |
| Ferrovibrio | 0,000834 | 0,000156 | 0,000000 |
| Filomicrobium | 0,000060 | 0,000000 | 0,000000 |
| Hirschia | 0,008761 | 0,000000 | 0,000000 |
| Hydrogenophaga | 0,22723 | 0,00086 | 0,00216 |
| Hyphomicrobium | 0,001699 | 0,000000 | 0,000000 |
| Hyphomonas | 0,003636 | 0,000000 | 0,000000 |
| Ideonella | 0,00647 | 0,00000 | 0,00000 |
| Inhella | 0,00057 | 0,00000 | 0,00000 |
| Janthinobacterium | 0,00021 | 0,00070 | 0,00000 |
| Kaistia | 0,003010 | 0,000234 | 0,000000 |
| Lautropia | 0,00006 | 0,00000 | 0,00000 |
| Legionella | 0,00748 | 0,00008 | 0,00000 |
| Leptothrix | 0,00089 | 0,00008 | 0,00000 |
| Limnobacter | 0,00042 | 0,00000 | 0,00000 |
| Limnohabitans | 0,00149 | 0,00000 | 0,00000 |
| Malikia | 0,00021 | 0,00000 | 0,00043 |
| Massilia | 0,00027 | 0,00000 | 0,00000 |
| Mesorhizobium | 0,000745 | 0,000000 | 0,000000 |
| Methylocystis | 0,000328 | 0,000000 | 0,000000 |
| Methylophilus | 0,00015 | 0,00000 | 0,00000 |
| Methylotenera | 0,00218 | 0,00000 | 0,00000 |
| Methyloversatilis | 0,00015 | 0,00000 | 0,00000 |
| Neorhizobium | 0,000209 | 0,000000 | 0,000000 |
| Nevskia | 0,00176 | 0,00000 | 0,00000 |
| Nitrobacter | 0,000060 | 0,002576 | 0,000000 |
| Nordella | 0,000149 | 0,000000 | 0,000000 |
| Novosphingobium | 0,002146 | 0,000000 | 0,000000 |
| OM60(NOR5) clade | 0,00054 | 0,00000 | 0,00000 |
| Others/Unclassified | 0,02816 | 0,01335 | 0,00216 |
| Others/Unclassified | 0,06005 | 0,01764 | 0,02133 |
| Paucibacter | 0,00781 | 0,00078 | 0,00000 |
| Pedomicrobium | 0,001013 | 0,000117 | 0,000288 |
| Pelomonas | 0,00069 | 0,00000 | 0,00000 |
| Phaselicystis | 0,00012 | 0,00000 | 0,00000 |
| Phenylobacterium | 0,001579 | 0,000468 | 0,000432 |
| Piscinibacter | 0,00030 | 0,00000 | 0,00000 |
| Plesiomonas | 0,00000 | 0,00008 | 0,00000 |
| Polaromonas | 0,00048 | 0,00000 | 0,00000 |
| Polymorphobacter | 0,000060 | 0,000000 | 0,000000 |
| Polynucleobacter | 0,00018 | 0,00000 | 0,00000 |
| Pseudomonas | 0,01639 | 0,02736 | 0,02768 |
| Pseudorhodoferax | 0,00009 | 0,00000 | 0,00000 |
| Pseudorhodoplanes | 0,000715 | 0,000000 | 0,000000 |
| Pseudoxanthomonas | 0,00009 | 0,00000 | 0,00000 |
| Reyranella | 0,073904 | 0,000000 | 0,000432 |
| Rheinheimera | 0,00006 | 0,00000 | 0,00000 |
| Rhizobacter | 0,01001 | 0,00016 | 0,00000 |
| Rhodobacter | 0,001520 | 0,000429 | 0,000000 |
| Rhodoferax | 0,00095 | 0,00000 | 0,00000 |
| Rhodopseudomonas | 0,000089 | 0,000000 | 0,001730 |
| Rhodovastum | 0,000387 | 0,000000 | 0,000000 |
| Rickettsiella | 0,00009 | 0,00000 | 0,00000 |
| Roseomonas | 0,003308 | 0,000156 | 0,000000 |
| Salinimonas | 0,00018 | 0,00000 | 0,00000 |
| Sandarakinorhabdus | 0,000060 | 0,000000 | 0,000000 |
| Serratia | 0,00000 | 0,03313 | 0,10148 |
| Shewanella | 0,00000 | 0,38640 | 0,82096 |
| Sideroxydans | 0,00006 | 0,00000 | 0,00000 |
| Skermanella | 0,000238 | 0,000000 | 0,000000 |
| Sphaerotilus | 0,00006 | 0,00000 | 0,00000 |
| Sphingobium | 0,001043 | 0,000000 | 0,000000 |
| Sphingomonas | 0,000864 | 0,000000 | 0,000000 |
| Sphingopyxis | 0,011860 | 0,000000 | 0,000000 |
| Sphingorhabdus | 0,006467 | 0,000000 | 0,000000 |
| Stella | 0,001430 | 0,000000 | 0,000000 |
| Sulfuritalea | 0,00006 | 0,00000 | 0,00000 |
| SWB02 | 0,000685 | 0,000000 | 0,000000 |
| Tabrizicola | 0,000924 | 0,000000 | 0,000000 |
| uncultured Acetobacteraceae | 0,006586 | 0,000000 | 0,000000 |
| uncultured Burkholderiaceae | 0,01785 | 0,00000 | 0,00187 |
| uncultured Caulobacteraceae | 0,000060 | 0,000117 | 0,000000 |
| uncultured Desulfarculaceae | 0,00033 | 0,00000 | 0,00000 |
| uncultured Hyphomonadaceae | 0,001341 | 0,000000 | 0,000000 |
| uncultured Paracaedibacteraceae | 0,009208 | 0,000195 | 0,000000 |
| uncultured Reyranellaceae | 0,000775 | 0,000000 | 0,000000 |
| uncultured Rhizobiales Incertae Sedis | 0,004589 | 0,000000 | 0,000000 |
| uncultured Rhodanobacteraceae | 0,00036 | 0,00000 | 0,00000 |
| uncultured Rhodobacteraceae | 0,002622 | 0,000078 | 0,000000 |
| uncultured Rickettsiaceae | 0,000060 | 0,000000 | 0,000000 |
| uncultured Solimonadaceae | 0,00030 | 0,00008 | 0,00000 |
| uncultured Sphingomonadaceae | 0,000447 | 0,000000 | 0,000000 |
| uncultured Steroidobacteraceae | 0,00036 | 0,00000 | 0,00000 |
| Undibacterium | 0,00006 | 0,00000 | 0,00000 |
| Variovorax | 0,17582 | 0,00020 | 0,00000 |
| Yersinia | 0,00000 | 0,00074 | 0,00072 |

**Supplementary Figure 1.** Prototype and cutter L-MFCs. A, The PMMA tube was equipped with two caps of PVC with brass nozzles (to regulate liquids and gas exchanges) and mobile carbon electrodes (B).

**
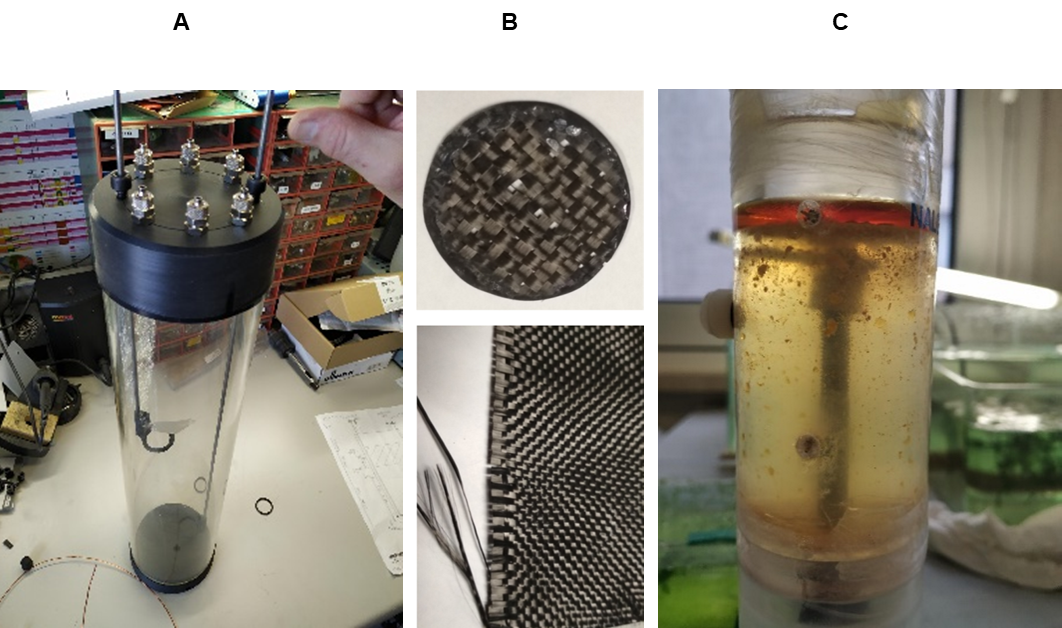
**
